# Comparison of the effects of simulated patient clinical skill training and student roleplay on objective structured clinical examination performance among medical students in Australia

**DOI:** 10.3352/jeehp.2019.16.3

**Published:** 2019-01-11

**Authors:** Silas Taylor, Matthew Haywood, Boaz Shulruf

**Affiliations:** 1 Office of Medical Education, University of New South Wales, Sydney, Australia; 2 Centre for Medical and Health Sciences Education, University of Auckland, Auckland, New Zealand; Hallym University, Korea

**Keywords:** Medical students, Patient simulation, Clinical competence, Communication, Volunteers, Australia

## Abstract

**Purpose:**

Optimal methods for communication skills training (CST) are an active research area, but the effects of CST on communication performance in objective structured clinical examinations (OSCEs) has not been closely studied. Student roleplay (RP) for CST is common, although volunteer simulated patient (SP) CST is cost-effective and provides authentic interactions. We assessed whether our volunteer SP CST program improved OSCE performance compared to our previous RP strategy.

**Methods:**

We performed a retrospective, quasi-experimental study of 2 second-year medical student cohorts’ OSCE data in Australia. The 2014 cohort received RP-only CST (N=182) while the 2016 cohort received SP-only CST (N=148). The t-test and analysis of variance were used to compare the total scores in 3 assessment domains: generic communication, clinical communication, and physical examination/procedural skills.

**Results:**

The baseline characteristics of groups (scores on the Australian Tertiary Admission Rank, Undergraduate Medicine and Health Sciences Admission Test, and medicine program interviews) showed no significant differences between groups. For each domain, the SP-only CST group demonstrated superior OSCE outcomes, and the difference between cohorts was significant (P<0.01). The superiority of volunteer SP CST over student RP CST in terms of OSCE performance outcomes was found for generic communication, clinical communication, and physical examination/procedural skills.

**Conclusion:**

The better performance of the SP cohort in physical examination/procedural skills might be explained by the requirement for patient compliance and cooperation, facilitated by good generic communication skills. We recommend a volunteer SP program as an effective and efficient way to improve CST among junior medical students.

## Introduction

Effective communication between doctors and their patients is recognised as a critical goal because of the wide-ranging benefits that have been shown to accrue to both participants from such interactions, including improvements in doctor and patient satisfaction and various measures of patient outcomes [1]. Programs of communication skills training (CST) and the assessment thereof are widely established as important components of medical curricula, particularly since effective communication is not necessarily an inherent skill or determined by personality, but can be both taught and learned [2]. Didactic methods of CST are now generally augmented by experiential learning approaches to CST, with medical programs tending to employ real patients, simulated patients (SPs), and student roleplay (RP) in various combinations for this purpose; the mix of techniques is determined by factors including the educational stage and needs of the student and resource availability [3]. A considerable amount of research has attempted to determine the optimal approach to CST, although objective comparisons of SP and RP methods are somewhat lacking [1]. The available evidence suggests that these methods are equivalent for teaching certain communication skills [4-6], although RP may be superior to SP in fostering empathy for patient perspectives because RP inherently requires students to assume the patient perspective [4]. In addition, RP is considered more cost-effective as a consequence of the student’s role as both trainer and learner [7]. While students do value the RP method and it can be educationally useful [8], it may not be preferred by participants and may limit learning opportunities [8]. Alternatively, a well person playing the role of a patient for the purpose of students learning clinical skills in controlled circumstances (namely, an SP) offers a potentially more acceptable approach to CST by providing students with objective feedback on their performance [1]. SP programs are favoured by both learners and faculty because they expose students to unfamiliar people and authentic, unbiased interactions with appropriately intense emotional elements in a safe and supported learning environment [9]. It should be noted that SP programs require significant administrative and financial investment, with trained professional medical actors receiving more than AUD 80 per hour (EUR 55, USD 60) [10]. As a result, SP programs utilising volunteers have been proposed as a more sustainable means to achieve the same teaching outcomes, and have been shown to be equally effective educationally to RP [11].

The assessment of communication skills involves real patients as well as SPs, and is generally delivered through objective structured clinical examinations (OSCEs) [12]. OSCEs have been established as the clinical assessment of choice in medicine, nursing, exercise physiology, physiotherapy, and other allied health programs and commonly examine multiple skillsets simultaneously. As such, OSCEs can generate scores for generic communication skills (how the student forms and maintains a relationship with the patient and understands their perspective) and clinical communication skills (medical history-taking).

Given the available evidence regarding the optimum CST approach, our institution (the University of New South Wales) implemented an inexpensive volunteer SP program in 2015 that replaced our pre-existing student RP program and aimed to deliver improved pre-clinical CST. Two years after the SP program was first implemented, the present study set out to determine whether this volunteer SP method translated into improved OSCE performance in communication skills compared to our previous RP strategy. An OSCE at the end of year 2 forms a barrier to progression into the clinical phase of our program. Due to the relative reliability and validity of OSCEs as an assessment method, these circumstances provided the opportunity to compare 2 forms of CST as objectively as possible. To our knowledge, this is the first study of its kind in an Australian undergraduate medical program.

## Methods

### Ethical statement

This study was granted ethical approval by the University of New South Wales Human Research Ethics Committee (Ref: HC15421). Informed consent was obtained from participants.

### Materials and subjects

Volunteer SPs were drawn from the local community and given support to play realistic patient roles at our clinical skills centre using pre-written scenarios. The cohort of students who engaged in RP enrolled onto the medicine program in 2013 and took an OSCE at the end of their second pre-clinical year in 2014; similarly, the SP cohort of students enrolled in 2015 and were examined in 2016. Clinical skills sessions occurred in 5 clinical courses relating to organ systems (cardiovascular, respiratory, gastro-renal, neurological, and musculoskeletal) and 2 discipline-specific courses (paediatrics/maternal health and dermatology). These sessions took place over the duration of year 1 and 2 in addition to other course content, which was largely identical for both groups and included lectures, scenario group learning, and bedside teaching in public hospitals. Each student participated in 3 campus-based clinical skills sessions per course, with the last session of each course dedicated to developing communication skills utilising RP or SP interactions relevant to the course content.

In the OSCE at the end of year 2, students were evaluated against our institution’s established assessment criteria, which were presented to examiners as electronic forms in an app (Table 1). The OSCE stations set in 2014 and 2016 for the 2 cohorts of students were identical. The assessment comprised six 15-minute stations, each manned by 1 examiner and containing tasks related to all 3 clinical skills domains (i.e., generic communication skills, clinical communication skills, and examination/procedural skills). Each station therefore required candidates to take a history (clinical communication skills), perform an examination or procedural skill (examination/procedural skills), and provide an explanation or advice to the patient (generic communication skills). In total, 6 separate OSCE sittings across 4 clinical sites were conducted for each cohort. Each sitting comprised a unique set of stations not replicated in any other sitting for that cohort, such that 36 unique stations were used across the 6 exams. In order to pass the assessment, students were required to achieve a minimum total score in each of the 3 clinical skills domains across the whole OSCE.

Each criterion in each OSCE station was graded as fail (F), borderline (P-), clear pass (P), or pass with distinction (P+), as per the standard medicine program marking guidelines at our institution (Table 2). Examiners were given the option to provide justifications for their grades by either selecting a generic reason from a pre-prepared list or entering their own free-text response. Post-exam processing assigned a numerical value to each grade (F=3, P-=5, P=7, and P+=9), and the numerical values for the relevant criteria for each domain from all OSCE stations were then totalled for each individual student. Ultimately, each student received 4 marks: 3 representing the total score across the whole OSCE for each of the domains, and 1 aggregate mark of the 3 domain scores.

All examiners were clinicians familiar with the examination format and had received identical OSCE assessment training in the form of reading material and a verbal briefing. Admission data for examined students were extracted from electronic university records and included the number of students in each cohort, admission category breakdown, and scores for the Australian Tertiary Admission Rank (ATAR), Undergraduate Medicine and Health Sciences Admission Test (UMAT), and medicine program interview. OSCE data were retrieved from electronic records and included the student identification number, examination date/site/time, grades awarded, and corresponding numerical values for each criterion at each station and total marks for the 3 domains, as described above. This study included data from a total of 330 students who sat the OSCE in 2014 (N=182) and 2016 (N=148) and for whom there was a complete admission dataset (UMAT, ATAR, and interview score), ensuring comparability. These full datasets were available only for domestic school leavers who were admitted via the mainstream admission path. All other students had been admitted via different admission paths (e.g., international, indigenous, or graduate-entry) using different selection tools and were therefore excluded from the present analysis. This exclusion removed some potentially significant confounders from the data and allowed the data to be analysed without missing values.

### Technical information

A retrospective, quasi-experimental approach was used to study the OSCE performance of students who had participated in RP-only CST compared with students who had participated in SP-only CST.

### Statistics

The statistical analysis was performed using IBM SPSS ver. 24.0 (IBM Corp., Armonk, NY, USA). The t-test and analysis of variance were used to compare the raw and the estimated marginal means (EMM) of students’ total scores for generic communication (criteria 1, 3, and 9 in Table 1), clinical communication (criteria 4, 5, and 6 in Table 1) and physical examination/procedural skills (criteria 7 and 8 in Table 1), adjusted for age and grouped by cohort (2014 or 2016). The t-test (2-tailed) with assumption of equal variance was applied to the data on baseline characteristics and EMM scores to evaluate the presence of significant differences between the 2 cohorts. Post hoc multiple comparison analysis using the Fisher least significant difference was used to assess differences in EMM scores. P-values <0.05 were considered to indicate statistical significance.

## Results

The results showed that the 2016 cohort was not statistically significantly different from the 2014 cohort in terms of the results of selection tools, with the exception of UMAT scores, which were lower for the 2016 cohort than for the 2014 cohort (62.61 versus 64.39). However, OSCE scores grouped by domain and overall score were significantly higher for the 2016 cohort compared to the 2014 cohort (Table 3). The effect sizes of the differences were moderate (0.37–0.44). Further, univariate analysis of variance was performed to identify EMMs by cohort, while controlling for the impact of scores on the admission tools. The results show that the impact (Cohen’s d) was slightly decreased but remained within the low-moderate effect size range (0.26–0.34), with statistical significance (P<0.01) (Table 4). The raw data are available in Supplement 1.

## Discussion

Our volunteer SP program for CST had a significant positive impact on students’ generic and clinical communication performance on OSCEs compared to the previous and commonly employed RP approach. Interestingly, this difference between RP and SP cohorts was maintained in the physical examination/procedural skills domain. A simple, transparent statistical analysis indicated that despite the 2016 cohort scoring lower on the UMAT (P=0.014) and ATAR (not significant), they performed better on the OSCE, a finding that further strengthens our assertion regarding the impact of SP. Our findings can be attributed with reasonable confidence to our SP program intervention alone, thus advancing our understanding of optimal methods for CST in preclinical medical students, an important area of medical education research [1]. These results appear to refute other work that has thus far suggested either equivalence of RP and SP [13] or superiority of the former strategy [4]. Importantly, the transition from RP to volunteer SP CST has been a cost-neutral process at our institution, in contrast to previous comparative analyses of professional SP and RP CST that have demonstrated inferior cost-effectiveness of the SP method [7]. While the authors acknowledge the established benefits of increased empathy and understanding of the patient’s perspective through RP [4], we argue that there are many clear advantages to using volunteer SPs that are not captured by structured observer rating forms. For example, the mean age of our SP pool (57.7 years old) is such that many are very experienced real patients who can draw on countless interactions with healthcare professionals to inform both their SP persona and their impressions of the student’s performance. This in turn imbues their feedback with an authentic patient perspective, while RP can only provide an ‘appreciation’ of that perspective (i.e., empathy) as a construct entirely dependent on the student’s own perceptions and biases. Additionally, students have reported that SP feedback is more instructive and provides specific indicators that they can act on to improve their communication skills, while the interaction itself tends to be more engaging due to heightened emotional intensity. Learners also appreciate the opportunity to practice their skills with SPs before encountering real patients for the first time [14]. Finally, we have noticed that SP feedback is more oriented towards non-technical skills than peer feedback, a focus that we consider to be the most effective use of contact teaching time for CST. In contrast, students are generally familiar with the role of the doctor and medical terminology, and therefore are not as challenged in the RP setting when engaging with peers to use patient-appropriate language and behaviour.

The unexpected result of improved OSCE scores in the domain of physical examination/procedural skills for the SP cohort may be explained in terms of the benefits that effective communication skills can bring to overall clinical performance. In particular, enhanced consultation effectiveness and efficiency, higher satisfaction, and an improved therapeutic relationship may be experienced by both patient and doctor in this setting [1]. Whilst performing physical examinations and conducting procedures on patients may at first glance appear to be related purely to technical proficiency, these tasks do require patient compliance and cooperation. Hence, good generic communication skills (including non-verbal communication skills) can be expected to facilitate improved performance beyond this domain, through establishing a strong rapport that enables physical examinations to be conducted sensitively and fluently.

A limitation of this study may lie in the equivalency of the 2 cohort groups according to demographic information. However, it should be noted that there were no significant differences according to characteristics such as age (P=0.204) and ATAR scores (P=0.59) between the 2 cohorts. Whilst the UMAT score (P=0.014) showed a significant difference between the 2 groups, as mentioned above, the 2016 cohort, which scored lower on the UMAT (P=0.014), performed better on the OSCE.

In conclusion, we have presented evidence to suggest that CST using volunteer SPs produces superior results in generic communication, clinical communication, and physical examination/procedural skills when compared to RP CST. Our volunteer SP program created no additional financial burden compared to the previous RP approach, an important consideration for any institution for whom paid professional actors would outstrip the resources available for CST. These findings add a counterpoint to some studies in the current literature and suggest that a volunteer SP program can contribute significantly to CST programs among junior medical students.

## Figures and Tables

**Table 1.** Objective structured clinical examination assessment criteria for University of New South Wales Medicine program

Assess the student's ability to:	Mark–circle one grade for each			
1. Initiate and end the consultation: greet patient, introduce self, outline agenda, seek permission to proceed, thank the patient, and offer help with repositioning, dressing, etc.	F	P-	P	P+
	Fail	Borderline	Pass	Exceptional
2. Listen attentively, engage patient, and maintain respect: allow patient to use his or her own words without premature interruption, use open and closed questions, reflect important feelings, pick up verbal and non-verbal cues, display sensitivity to patient’s needs, respect boundaries, and gain patient’s trust	F	P-	P	P+
	Fail	Borderline	Pass	Exceptional
			
3. Elicit a relevant clinical history: establish reason for presentation, course and nature of symptoms; summarise patient’s symptoms to check understanding	F	P-	P	P+
	Fail	Borderline	Pass	Exceptional
4. Elicit a psychosocial history: ask patient about relevant family, social support, cultural, lifestyle factors, employment issue, as appropriate	F	P-	P	P+
	Fail	Borderline	Pass	Exceptional
5. Gather relevant past medical and family history: ask about past personal and family history, as well as specific risk factor history where appropriate	F	P-	P	P+
	Fail	Borderline	Pass	Exceptional
6. Communicate with patient and ensure patient comfort when conducting a physical examination/skill: explain to patient what is being done, provide suitable instructions, and ensure the patient’s privacy and comfort	F	P-	P	P+
	Fail	Borderline	Pass	Exceptional
7. Perform technically competent physical examination or skill (1): correct positioning of patient, adept with equipment, competent approach to examination	F	P-	P	P+
	Fail	Borderline	Pass	Exceptional
8. Perform technically competent physical examination or skill (2): correct positioning of patient, adept with equipment, competent approach to examination	F	P-	P	P+
	Fail	Borderline	Pass	Exceptional
9. Summarise case findings: should use medical jargon, identify patient’s key concerns and reason for presenting and summarise relevant history and examination findings	F	P-	P	P+
	Fail	Borderline	Pass	Exceptional

**Table 2.** Adapted generic assessment descriptors used in the University of New South Wales Medicine program

Grade	F	P-	P	P+
Explanation of grade	The student misunderstood the assessment requirements, or failed to address the most important aspects. This grade represents a clear and substantial failure.	Addresses the assessment criteria at a standard that is barely satisfactory for students at that stage of the program. This grade represents a low or conceded pass.	Addresses the assessment criteria at a standard that is satisfactory for students at that stage of the program. One or two aspects may not be well done, but the standard is still considered to be satisfactory and this grade covers both pass and credit performances	Addresses the assessment criteria at a standard that exceeds what is normally considered satisfactory for students at that stage of the program—high distinction.

**Table 3.** Comparison of cohorts by admission and OSCE scores (t-test)

Variable	Year	No. of participants	Mean± standard deviation	SE	Significance	Mean difference	t-value	SE difference	95% Confidence interval	Cohen's d
Age (yr)	2014	182	19.87±0.74	0.055	0.204	0.14	-1.363	0.103	-0.342 to 0.062	0.19
	2016	148	20.01±1.12	0.092						
Undergraduate Medicine and Health Sciences	2014	182	64.39±7.19	0.533	0.014	-1.78	2.481	0.715	0.368 to 3.183	-0.25
Admission Test	2016	148	62.61±5.80	0.477						
Australian Tertiary Admission Rank	2014	182	99.15±1.51	0.112	0.059	-0.37	1.895	0.197	-0.014 to 0.759	-0.25
	2016	148	98.78±1.96	0.161						
Interview	2014	182	87.01±9.72	0.720	0.713	0.41	-0.368	1.107	-2.585 to 1.770	0.04
	2016	148	87.42±10.34	0.850						
Generic Comm	2014	182	73.85±4.61	0.342	0.001	1.69	-3.394	0.498	-2.669 to -0.710	0.37
	2016	148	75.54±4.35	0.358						
Clinical Comm	2014	182	72.53±4.68	0.347	<0.0001	2.08	-4.317	0.483	-3.033 to -1.134	0.45
	2016	148	74.62±3.93	0.323						
Physical examination/procedural skills	2014	182	70.64±5.52	0.409	0.001	1.98	-3.378	0.587	-3.138 to -0.828	0.36
	2016	148	72.62±5.03	0.413						
OSCE total	2014	182	72.70±4.26	0.316	<0.0001	1.89	-4.169	0.452	-2.776 to -0.996	0.44
	2016	148	74.58±3.86	0.317						

OSCE, objective structured clinical examination; SE, standard error; Comm, communication.

**Table 4.** OSCE scores (total and by domain) by cohort (estimated marginal means)

Variable	Year	Mean±standard error	95% Confidence interval	Cohen's d
Generic Comm	2014	73.93±0.328	73.28-74.57	0.26
	2016	75.45±0.365	74.73-76.17	
Clinical Comm	2014	72.57±0.326	71.92-73.21	0.34
	2016	74.58±0.362	73.87-75.29	
Physical examination/procedural skills	2014	70.67±0.395	69.89-71.44	0.28
	2016	72.64±0.448	71.76-73.52	
OSCE total	2014	72.75±0.302	72.15-73.34	0.32
	2016	74.52±0.336	73.86-75.18	

The covariates appearing in the model were evaluated with the following values: age (yr)=19.93, Undergraduate Medicine and Health Sciences Admission Test=63.61, Australian Tertiary Admission Rank=98.98, and interview=87.23. OSCE, objective structured clinical examination; Comm, communication.

## Supplementary materials

Supplement 1. Data files are available from https://doi.org/10.7910/DVN/DO34NS

Supplement 2. Audio recording of the abstract.
